# Musculoskeletal impairment among Syrian refugees living in Sultanbeyli, Turkey: prevalence, cause, diagnosis and need for related services and assistive products

**DOI:** 10.1186/s13031-021-00362-9

**Published:** 2021-04-20

**Authors:** Dorothy Boggs, Oluwarantimi Atijosan-Ayodele, Hisem Yonso, Nathaniel Scherer, Timothy O’Fallon, Gülten Deniz, Selin Volkan, Ahmed Örücü, Isotta Pivato, Ammar Hasan Beck, İbrahim Akıncı, Hannah Kuper, Allen Foster, Andrea Patterson, Sarah Polack

**Affiliations:** 1 International Centre for Evidence in Disability, London School of Hygiene & Tropical Medicine, Maidstone, UK; 2 Maidstone and Tunbridge Wells NHS Trust, Maidstone, UK; 3 Relief International, Istanbul, Turkey; 4 Mülteciler Derneği, Istanbul, Turkey; 5 International Centre for Eye Health, London School of Hygiene & Tropical Medicine, London, UK

**Keywords:** Population, Prevalence, Musculoskeletal impairment, Syrian refugee, Survey, Services, Assistive products (AP)

## Abstract

**Background:**

Epidemiological data on musculoskeletal impairment (MSI) and related service and assistive product (AP) needs for displaced populations are lacking. This study aimed to estimate the prevalence, aetiology, and specific MSI diagnosis and the need for related services and APs among Syrian refugees living in Sultanbeyli, a district in Istanbul, Turkey.

**Methods:**

A population-based survey used probability proportionate to size and compact segment sampling to select 80 clusters (‘street’) of 50 individuals (aged 2+), for total sample size of approximately 4000 participants. An updated version of the Rapid Assessment of MSI tool (RAM) was used to screen all participants using six questions. Any participant who screened positive underwent a standardised examination by a physiotherapist to assess the presence, aetiology, severity and specific diagnosis of MSI and an assessment of need for related services and APs.

**Results:**

The all-age prevalence of MSI was 12.2% (95% CI 10.8–13.7) and this increased significantly with age to 43.8% in people 50 and older. Over half (51%) of MSI was classified as moderate, 30% as mild and 19% as severe. The war in Syria was identified as the direct cause for 8% of people with MSI. The majority (56%) of MSI diagnoses were acquired non-traumatic causes. There was high unmet need for rehabilitation services; for example, 83% of people with MSI could benefit from physiotherapy but were not receiving this service. Overall, 19% of people with MSI had an unmet need for at least one AP. Apart from availability of walking sticks/canes, coverage was low with less than half the people with MSI who needed APs and services had received them. The most common reasons for not seeking services and APs were ‘need not felt’, lack of service availability and of awareness of services, and financial barriers.

**Conclusions:**

MSI is common among the Syrian refugee population living in Sultanbeyli District, particularly older adults, however less than half have been able to access relevant services and APs. These findings can inform the planning of health services for migrant populations, including the essential integration of rehabilitation and APs, and increase access to these vital services.

**Supplementary Information:**

The online version contains supplementary material available at 10.1186/s13031-021-00362-9.

## Background

Epidemiological population-based data on musculoskeletal impairment (MSI) and the need for related services and assistive products (APs) are limited in low- and middle-income countries (LMIC) despite evidence that MSI-related difficulties are common [1–3]. In the *World Health Survey*, difficulties with mobility and pain were amongst the most commonly reported functional difficulties for adults aged 18 years and older*,* with more than 16.5% of respondents reporting mild or greater difficulty with ‘moving around’ [3, 4].

MSI data are particularly lacking for refugee populations despite increasing recognition of and commitment to disability inclusion in humanitarian contexts [5, 6]. A survey among Syrian refugees in Lebanon and Jordan found that 14.4% of adults reported difficulties walking, however these data were based on self-report only and may not capture all functional limitations related to MSI [7]. Conflict and displacement can increase the risk of impairment and disability either directly, such as new trauma and injuries related to war, especially in the context of disrupted health services, or indirectly, such as through the breakdown of infrastructure and social structures and loss/damage of APs. These risks may be especially common in situations of displacement where there can be varying levels of access to health and social care in host countries, which further cause and/or exacerbate impairments [8]. Data on MSI are needed in order to inform and advocate for services to maximise functioning, participation and quality of life among marginalised refugee populations [9].

MSI can result from many different health conditions, such as neurological, musculoskeletal, developmental and pain related conditions [including more than 150 of the 350 Global Burden of Disease (GBD) health conditions]; MSI assessment is therefore complex [1, 10, 11]. The Rapid Assessment of Musculoskeletal Impairment (RAM) is a validated clinical impairment screening tool developed by Oxford University and the International Centre for Evidence in Disability (ICED) to estimate population-based prevalence, aetiology and diagnoses of MSI [12]. It uses a two step-process which includes six initial screening questions to assess self-reported difficulties with the musculoskeletal system, followed by a clinician-led examination. The RAM [12] has been used in Rwanda, Cameroon and India where all age prevalence of MSI was found to be 5.2%, 11.6 and 19.6%, respectively [12–15]. Experience of using the RAM in these settings has identified a need to review and update the methodology including the screening questions, the method for assigning presence and severity of MSI, and the data collection on service and AP needs to improve utility of the data for health and rehabilitation service planning.

Estimates suggest that Turkey hosts 64% of Syrian refugees, totalling more than 3.6 million people [16]. The vast majority (96%) live among host communities in urban, peri-urban and rural areas [16]. Specifically, at the time of this study, approximately 20,000 Syrian refugees lived in the Sultanbeyli District, a sub-urban area on the outskirts of Istanbul hosting the largest number of refugees in a single district on the Anatolian side of the city [17]. Data on MSI and associated service needs among this displaced population are lacking, which hinders evidence-based advocacy and planning of services for this population. Using an updated version of the RAM tool, this study aims to estimate the prevalence, aetiology and diagnoses of MSI and the need for related services and APs among Syrian refugees living in Sultanbeyli.

## Methods

### Sampling

The study was conducted as part of a wider population-based survey of disability during August to October 2019 in Sultanbeyli District in Istanbul, Turkey. Based on previous surveys, an all-age [disability and] MSI prevalence was conservatively estimated to be 5%. Thus, a sample size of 4000 people aged 2 years and above was required, allowing precision of 20% around the estimates, 95% confidence, 20% non-response, and a design effect of 1.7.

Multi-stage cluster randomised sampling was used to select study participants. The municipality refugee registration database provided by Mülteciler Derneği, a local non-government organisation providing migrant social and healthcare services for refugees, was used as the sampling frame [18].

.A “cluster” was defined as a street within Sultanbeyli and 80 clusters were randomly selected using probability proportionate to size sampling. Within each cluster, households were randomly selected until at least 50 participants aged 2+ were included. When a street did not contain 50 participants, connecting and adjacent streets were randomly selected until the target number was achieved. For the purposes of this survey, all Syrians aged 2+ within selected households were included in the survey, regardless of ‘Temporary Protection’ status. To maximise the response rate: i) enumeration teams telephoned households in advance when possible to inform them of the survey and arrange a suitable time to visit; ii) at least two repeat visits were attempted if not available; and iii) revisits were scheduled over the phone when possible for weekday evenings and weekends.

### RAM methodology and adaptations

Building upon lessons learned from previous surveys, the RAM [12] underwent review by a development team of experts in MSI and population-based surveys to address the identified gaps. This section will give an overview of the RAM methodology highlighting the updates/revisions made with RAM tool version 2 provided in Additional file 1.

**Fig. 1:**
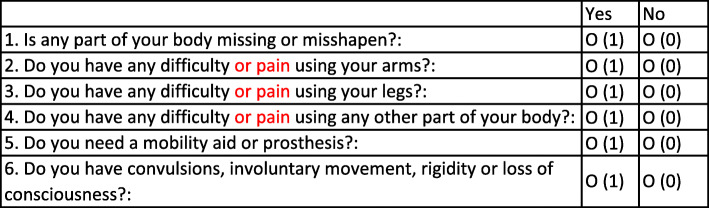
Rapid Assessment of Musculoskeletal six screening questions with update changes in red

The RAM tool consists of two stages. Six screening questions ask about difficulty using the limbs or body, use of AP, or experiences of convulsions or loss of conscience. Participants screen positive if they report yes to any of the questions, with a duration longer than one month or believed to be permanent. Based on existing MSI/pain research [2] and RAM findings in India [14], three of the screening questions were updated to include ‘pain’ in addition to ‘difficulty using’ the musculoskeletal system (see Fig. 1).

Anyone who screens positive then undergoes a standardised assessment by a physiotherapist and a physical examination and observation of activities to assess aetiology, severity of impairment, specific diagnosis and related service and APs needs/unmet needs [12].

First, participants undergo a standardised observation of four sets of activities to assess body functioning and examination of the structure of the affected area. The four sets of activities involve: i) positioning with squat to stand raising both arms straight over head; ii) mobility by walking along a 11-m rope in less than 10 s with or without limping; and iii) right and iv) left upper limb function by touching nose and picking up a coin to put in cup and tip into bowl. These observations, assessed in the previous version of RAM using a binary can/can’t response, were revised to a graded response: can do easily, can do with difficulty and cannot do.

Second, participants are asked about the timing and aetiology of the impairment and an examination of the affected structure is conducted. In the revised RAM, this section of the tool was simplified from 23 individual body items to five categories of main body areas, with individual items listed within the respective body area grouping. In the previous RAM, data were also collected on the nature of change and magnitude, however these sections were omitted in the revised version as they were considered redundant based on analysis of previous surveys.

**Fig. 2:**
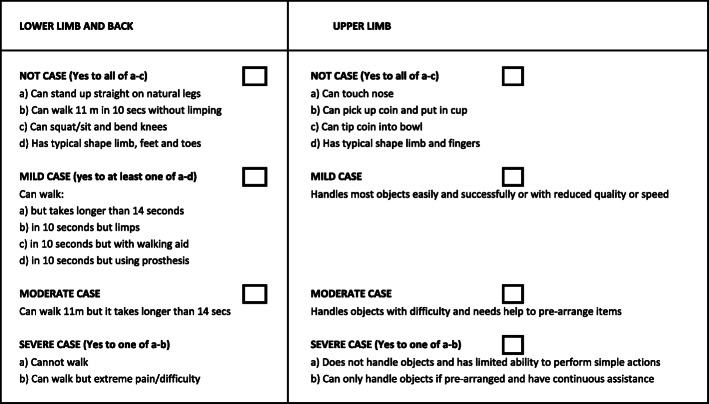
Rapid Assessment of Musculoskeletal case severity card

Third, based on these interviews and examinations, the participant is then categorised by the physiotherapist as having “no” MSI or a “mild”, “moderate” or “severe” MSI with respect to the musculoskeletal system’s ability to function. In the revised version we developed specific definitions (previously lacking) for these categories to ensure greater consistency within and between surveys (see Fig. 2).

Fourth, the physiotherapist assigns a specific diagnosis within the five clinical categories (congenital, infective, traumatic, acquired non-traumatic or neurological). Up to a maximum of three diagnoses per case could be assigned.

Fifth, participants are asked about their past/current use of services, including treatment or rehabilitation, and APs. Physiotherapists then make referral recommendations based upon their clinical judgement. This section of the tool was updated to include more detailed and structured questions to better inform identification of service and AP needs.

Finally, the tool was programmed using Open Data Kit (ODK) so data could be collected using mobile tablets.

### Data collection

Data collection tools were forward and back translated into Arabic to assess for accuracy and conceptual equivalence and pilot tested with members of the target population.

In each cluster, all eligible survey participants (aged 2+) were documented by an enumerator who then administered the six screening questions for MSI. Participants who screened positive with the questionnaire were visited, at their home, by a trained Syrian physiotherapist who knew the language, either the next day or a later date as convenient for the participant. The physiotherapist re-administered the six initial screening questions and then conducted the RAM as described above. For those cases of MSI for which ‘no specific diagnosis’ was recorded, their assessment data were reviewed by three research clinician authors (DB, TO, OA) who by consensus agreed and recorded specific diagnoses.

Data collection took place in the participant’s own homes. A proxy response was provided by a primary caregiver for children aged 2–10 or for any participants unable to communicate independently, in the presence of the participant where possible.

Survey data were collected on android tablets using LSHTM’s ODK software. Data on each tablet was encrypted and uploaded at the end of each day via Wi-Fi to a secure, password-protected, cloud-based server.

### Training

The wider disability survey was completed by four teams who underwent ten days of training, which included three days field pilot. Three physiotherapists conducted the RAM. The physiotherapists’ five-day classroom training was led by authors (OA, DB and HY) with lectures, role plays, discussions and observed practise assessments with patients at a physiotherapy centre. Training included physiotherapists independently completing assessments for the same participant to develop inter-rater agreement.

### Data analysis

Data were analysed using STATA 16.0 (StataCorp LP, College Station, Texas). The ‘svy’ command was used to derive proportion estimates accounting for cluster sampling.

We calculated proportions for each service and APs to determine, if ever received, current access and location, unmet need and for reasons for not seeking the service/AP.

### Ethical approval

Ethical approval for the study was provided by: London School of Hygiene & Tropical Medicine Observational Ethics Committee; Istanbul Sehir Univesity Research Ethics Committee; and Republic of Turkey Ministry of Interior: Directorate General of Migration Management.

Informed consent (written or thumbprint) was initially sought from self-identified heads of each household and subsequent consent was sought from all adult household participants who took part in the population-based survey. For participants under the age of 18 or for adults unable to communicate, verbal assent was sought from the participant using a simplified information sheet and written consent was sought from a parent or caregiver.

All participants identified in the survey as having health needs, including rehabilitation and APs, were referred to relevant local services which had been previously identified.

## Results

**Fig. 3:**
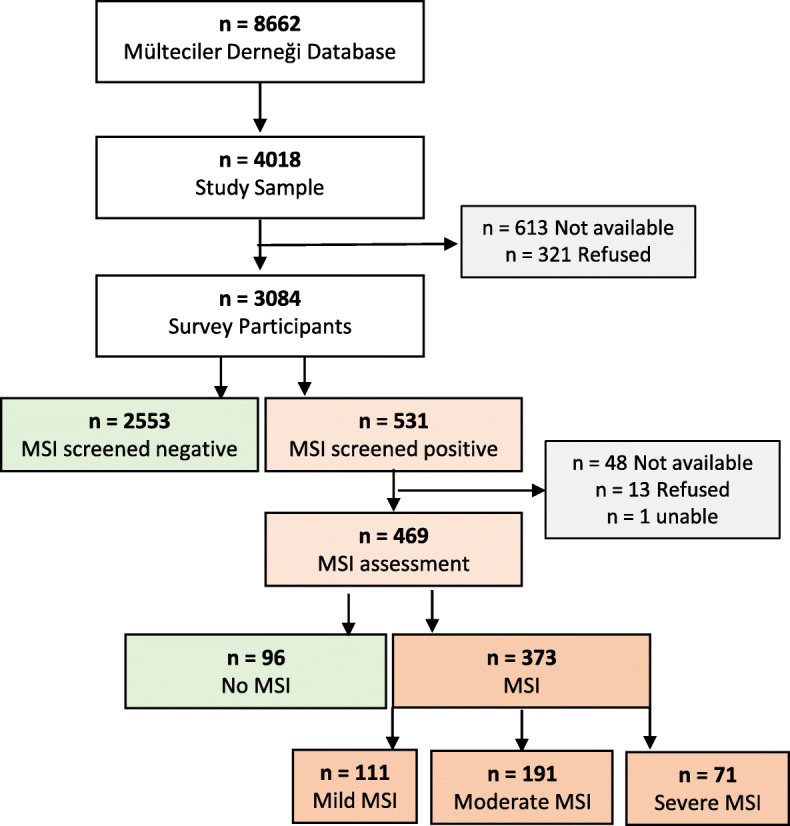
Sultanbeyli musculoskeletal survey participant flow chart

Of 4018 eligible participants, 3084 participated in the survey (response rate of 77%). In total, 613 (15%) were unavailable and 321 (8%) refused to participate. Compared to those who took part in the survey, non-participants were, more likely to be male (47% vs 65%, *p* < 0.001). The response rate was slightly lower among adults aged 18–49 (72%) and 50+ (75%) compared to children (82%), *p* < 0.001. Out of the 531 people who screened positive for MSI, 470 (89%) underwent MSI assessment, 48 (9%) were unavailable, 13 refused (2%) and 1 (< 1%) was unable to participate. Of the 469 participants who were assessed, 373 were confirmed to have MSI and 96 participants who screened positive were assessed not to have an MSI (see Fig. 3).

As shown in Table 1, the age and sex distribution of the study population was similar to that of the full population of registered refugees in Sultanbeyli. The study population was relatively young; 50% were under 20 years and only 3% were aged 60+ years.

**Table 1:** Age and gender distribution of district (database) and study sample population

	Total				Males				Females			
	Registration database		Study sample		Registration database		Study sample		Registration database		Study sample	
**Age (years)**	N	%	N	%	N	%	N	%	N	%	N	%
**2–9**	4793	26%	875	28%	2497	26%	442	31%	2296	26%	432	26%
**10–19**	4440	24%	773	25%	2316	24%	372	26%	2124	24%	401	24%
**20–29**	3558	19%	509	16%	1735	18%	199	14%	1823	20%	310	19%
**30–39**	2844	15%	446	14%	1574	16%	207	14%	1270	14%	239	15%
**40–49**	1545	8%	239	8%	795	8%	107	7%	750	8%	132	8%
**50–59**	935	5%	161	5%	484	5%	78	5%	451	5%	83	5%
**60+**	547	3%	81	3%	267	3%	38	3%	280	3%	43	3%
**Total**	18,662	100	3084	99	9668	100	1443	100	8994	100	1640	100

### Prevalence of MSI

In total, 373 of the 3022 survey participants were identified as having an MSI with overall prevalence of 12.2% (95% CI 10.8–13.7) (see Table 2). The prevalence increased by age from 3.9% (95% CI 3.0–5.1) in children (2–17 years) to 43.8% (95% CI 37.0–50.9) among adults aged 50+ years (*p* < 0.001). In terms of severity, 30% of MSI cases were mild, 51% moderate and 19% were severe. The overall prevalence of moderate or severe impairment was 8.6% (95% CI 7.5–9.8) and was 14.2% (95% CI 12.3–16.2) in adults aged 18 years and older. The prevalence of mild MSI was higher in females (4.7%, 95% CI 3.5–6.2, *p*-value 0.002) than males (2.5% 95% CI 1.7–3.6), but there was no significant difference in the prevalence of moderate or severe MSI.

**Table 2:** Prevalence of musculoskeletal impairment by age, gender and impairment severity

	TOTAL		2–17		18–34		35–49		50+ years		Male		Female	
	N	% (95% CI)	N	% (95% CI)	N	% (95% CI)	N	% (95% CI)	N	% (95% CI)	N	% (95% CI)	N	% (95% CI)
**Mild**	111	3.7 (2.8–4.7)	11	0.7 (0.4–1.4)	37	4.4 (3.0–6.4)	33	7.7 (5.5–10.6)	30	12.8 (8.9–18.1	35	2.5 (1.7–3.6)	76	4.7 (3.5–6.2)
**Moderate**	191	6.2 (5.3–7.3)	30	1.9 (1.3–2.8)	43	4.9 (3.5–6.7)	62	14.7 (11.4–18.6)	56	23.8 (18.8–29.7)	90	6.2 (5.0–7.7)	101	6.2 (4.9–7.9)
**Severe**	71	2.3 (1.8–3.0)	19	1.3 (0.8–2.0)	20	2.3 (1.4–3.6)	15	3.6 (2.2–5.9)	17	7.2 (4.4–11.8)	38	2.6 (1.9–3.6)	33	2.1 (1.4–2.9)
**All MSI**	373	12.2 (10.8–13.7)	60	3.9 (3.0–5.1)	100	11.5 (9.0–14.6)	110	26.0 (21.8–30.6)	103	43.8 (37.0–50.9)	163	11.4 (9.7–13.3)	210	13.0 (11–15.2)

Extrapolating the MSI prevalence to the estimated total population of 20,000 Syrian refugees living in Sultanbeyli suggests there are approximately 2560 people with an MSI, and 1790 would have with moderate or severe impairment.

### Aetiology

As shown in Table 3**,** trauma was the most common identified aetiology (16%) of MSI. Specifically, the war in Syria was identified as the direct cause for 8% of people with MSI. Developmental or nutritional causes were assigned as the aetiology for 11% of people with MSI. For over 25% of people the aetiology could not be identified.

**Table 3:** Aetiology of musculoskeletal impairment cases

Causes	Total causes^a^	
	N	%
**Family history**	**7**	**2%**
**Congenital but no family history**	**31**	**8%**
**Perinatal hypoxia**	**11**	**3%**
**Road traffic accident**	**13**	**4%**
**Trauma**^b^	**61**	**16%**
War in Syria	28	8%
Other war	2	0.5%
Deliberate self-harm	1	0.3%
Other accidents	30	8%
**Developmental / nutritional**	**42**	**11%**
**Infection**	**22**	**6%**
**Neoplasm**	**4**	**1%**
**Iatrogenic**	**2**	**0.5%**
**Unknown**	**96**	**26%**
**Other**^c^	**132**	**35%**
Herniated disc	57	15%

^a^Some participants had two causes so there were a total of 421 causes for 373 people

^b^A breakdown by type of trauma is provided

^c^A breakdown by ‘other’ is provided for herniated disc only (note: direct translation was herniated nucleus pulposus)

### Specific diagnoses

There were a total of 519 specific diagnoses for 373 participants with MSI (Table 4). Of the 519 MSI diagnoses over half (*n* = 291, 56%) were acquired non-traumatic causes, with spinal pain limiting function being the most common individual specific diagnosis. Nearly one-quarter (*n* = 123, 24%) of MSI diagnoses were acquired trauma, 10% (*n* = 53) were neurological, 1% (*n* = 6) were due to infection and 9% (*n* = 46) were congenital.

**Table 4:** Clinical diagnoses by type in 373 Syrian refugees with musculoskeletal impairment in Sultanbeyli, Turkey

Diagnosis	Number	Total in category^a^ N (%)
**A. Congenital**		**46 (9%)**
Other congenital hand deformity	1	
Other congenital abnormality of upper limb	6	
Developmental dysplasia of hip	4	
Proximal focal femoral deficiency	2	
Club foot	7	
Other congenital abnormality of lower limb	11	
Congenital deformity of cervical spine	2	
Congenital deformity of thoracolumbar spine	6	
Multiple congenital abnormalities	7	
**B. Infection**		**6 (1%)**
Joint infection	4	
Bone infection spine	2	
**C. Acquired traumatic**		**123 (24%)**
Fracture non-union	4	
Fracture malunion	7	
Spinal injury	7	
Head injury	3	
Recurrent/chronic dislocation	1	
Post traumatic joint stiffness	28	
Tendon problem	17	
Muscle problem	18	
Peripheral nerve problem	8	
Amputation	3	
Other trauma	27	
**D. Acquired non-traumatic**		**291 (56%)**
Degenerative joint disease	86	
Non-infective non-traumatic joint disease	20	
Bow legs	1	
Knock knees	2	
Skin/Soft tissue tumour	1	
Spinal deformity-kyphosis	2	
Spinal deformity-lordosis	1	
Spinal deformity-scoliosis	2	
Spinal pain limiting function	102	
TB spine/spine infection	1	
Limb pain limiting function	51	
Lymphoedema	1	
Other acquired non-traumatic	21	
**E. Neurological**		**53 (10%)**
Epilepsy	11	
Developmental delay	1	
Cerebral palsy - spastic	3	
Cerebral palsy - other	1	
Paraplegia	2	
Hemiplegia	3	
Peripheral nerve palsy	1	
Other neurological	31	
**TOTAL**	**519**	**519**

^a^Participants could have up to three diagnoses so there were a total of 519 diagnoses for 373 people

**Fig. 4:**
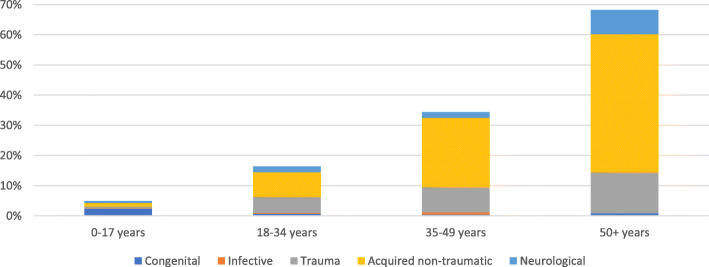
Clinical diagnostic categories of musculoskeletal impairment, by age group

Diagnoses varied by age (Fig. 4). The prevalence of congenital diagnoses was highest in children (2–17 years) at 2%, while neurological diagnoses was highest in the older age group 50 and older at 8%. Trauma related MSI increased with age from 0.7% among 0–17 years to 14% the > 50 years age group. The proportion of acquired non-traumatic diagnoses also increased substantially with age so that 46% of people with MSI aged > 50 years had this diagnosis.

### Service use and need

As shown in Table 5, overall service need, defined as people with MSI who were currently receiving/awaiting the service and those who (according to the physiotherapist) could benefit from a particular service but had not/were not currently receiving it, was high among people with MSI. Physiotherapy had highest service need (86%) among people with MSI, followed by medication (70%), information/exercises (40%), surgery (21%), other rehabilitation (15%), other services (13%) and environmental modifications (12%). Among the total survey population, 11% needed physiotherapy, 9% needed medication and 5% needed information/exercises, with all other assessed service need < 2.5%.

**Table 5:** Services for individuals with musculoskeletal impairment: need, access, unmet need and barriers

	Medication N (%)	Surgery N (%)	Physiotherapy N (%)	Information/exercises N (%)	Other rehabilitation^**+**^ N (%)	Environmental modifications N (%)	Other services N (%)
Overall service need* (MSI population *n* = 373)	260 (69.7%)	77 (20.6%)	322 (86.3%)	148 (39.7%)	53 (14.2%)	44 (11.8%)	47 (12.6%)
**Ever received service**	184 (49.3%)	66 (17.7%)	75 (20.1%)	25 (6.7%)	4 (1.1%)	6 (1.6%)	3 (0.8%)
**Ever received service in Turkey**	164 (44.0%)	29 (7.8%)	61 (16.4%)	21 (5.6%)	1 (0.3%)	2 (0.5%)	1 (0.3%)
**Currently receiving**^**++**^	121 (34.4%)	5 (1.3%)	14 (3.8%)	5 (1.3%)	–	2 (0.5%)	1 (0.3%)
Unmet service need** (MSI population n = 373)	139 (37.3%)	72 (19.3%)	308 (82.6%)	143 (38.3%)	53 (14.2%)	42 (11.3%)	47 (12.6%)
Coverage***	47%	6%	4%	3%	0%	5%	2%
**Reason not seeking service**							
Need not felt by participant	57.6%	38.9%	47.7%	62.9%	32%	23.8%	19.1%
Unaware of available services	15.8%	9.7%	38%	53.1%	25%	23.8%	31.9%
Could not afford	17.3%	26.3%	16.2%	16.8%	26.4%	41.9%	51%
Service not available	16.5%	31.9%	24%	30.8%	41.5%	40.5%	40.4%
Transport not accessible	2.2%	–	3.6%	1.4%	1.9%	2.4%	4.3%
Transport too expensive	3.6%	5.6%	11.4%	14.7%	7.5%	11.9%	10.6%
Service too far away	2.9%	2.8%	4.5%	6.3%	–	–	2.1%
Negative attitude of service providers	3.6%	8.3%	2.6%	0.7%	3.8%	–	6.4%
No translator	4.3%	8.3%	2.6%	1.4%	1.9%	–	2.1%
No one to accompany me	0.7%	–	0.6%	–	1.9%	2.4%	–
Other, please specify:	6.5%	11.1%	8.8%	1.4%	5.7%	–	4.2%

*Abbreviations*: ^**+**^Other rehabilitation included occupational therapy, speech and language therapy and psychosocial support; *Overall need = Need but not receiving + currently receiving/awaiting service; ^**++**^For surgery only, participants were asked ‘Currently seeing a surgeon or awaiting a surgical intervention?’; **Unmet service need = need but not receiving service; ***Coverage = (currently receiving/awaiting) / (Need but not receiving + currently receiving/awaiting)

The most commonly ever received services, among people with MSI, were medication (49%) followed by physiotherapy (20%) and surgery (18%). Specifically, in Turkey, the government hospital was the most commonly accessed service for medication (33% of those who had accessed services for medication) and surgery (100%). The Migrant Health Centre was most commonly used service for physiotherapy (79%), information/exercises (80%) and environmental modifications (50%).

Unmet need for services, defined as the proportion of people with MSI who (according to the physiotherapist) could benefit from a particular service but had not/were not currently receiving it, was high, with 347 of 373 (93%) people with MSI not receiving *at least* one service related to MSI that they could benefit from. This included 308 (82.6%) people with MSI who could benefit from physiotherapy, 143 (38.3%) people information/exercises, 139 (37.3%) medication, 72 (19.3%) surgery and 53 (14.2%) for other rehabilitation. No difference was found in unmet need for *at least* one service between males and females.

The reasons for not seeking services varied between service type; however, the most common reasons given were ‘need not felt’ (19% to 63%), lack of awareness of services (10% to 53%), financial barriers (16% to 51%) and lack of service availability (17% to 42%).

Applying estimates of unmet need to the overall study population suggests 10% of Syrian refugees living in Sultanbeyli need, but are not receiving physiotherapy, 4.7% information/exercises and 2.4% surgery. Overall, 11.5% (*n* = 347) of the study population needed but were not receiving *at least* one service related to MSI that they could benefit from. Extrapolating to the estimated total population of 20,000 Syrian refugees living in Sultanbeyli suggests there are approximately 2400 people who need, but are not be receiving *at least* one MSI-related service.

Coverage was calculated as the proportion of people who were receiving a service out of those who needed the service (i.e. those receiving a service plus those who needed but were not receiving that service). Coverage was relatively low: 47% of the 260 people who needed medication were receiving it, while this was < 10% for surgery, physiotherapy, information/exercises, environmental modifications and other services.

### Assistive product use and need

As shown in Table 6, overall AP need, defined as people with MSI who were currently using the AP and those who (according to the physiotherapist) could benefit from a particular AP but had not/were not currently using it, was much lower than service need among people with MSI. Protective footwear need was highest (7.2%), followed by stick/canes (4.3%), orthotics (3.8%), wheelchairs (3.8%) quad/tripod sticks (3.2%), with other AP need was < 2.4% There was no need for ramps. Among the total survey population, overall AP need was < 0.5% for each one of the APs assessed.

**Table 6:** Assistive products for individuals with musculoskeletal impairment: need, access, unmet need and barriers

	Wheel-chair	Crutches	Stick/Cane	Quad/Tripod stick	Walking frame	Rollator	LL Pros.	UL Pros.	Orth.	Protective footwear	Toilet/Shower chair	Grab bars
Overall AP need* (MSI population *n* = 373)	14 (3.8%)	1 (0.3%)	16 (4.3%)	12 (3.2%)	6 (1.6%)	9 (2.4%)	3 (0.8%)	1 (0.3%)	14 (3.8%)	27 (7.2%)	17 (1.9%)	7 (1.9%)
**Ever received or currently using**	7 (1.9%)	3 (0.8%)	14 (3.8%)	1 (0.3%)	1 (0.3%)	4 (1.1%)	2 (0.5%)	–	7 (1.9%)	6 (1.6%)	5 (1.3%)	1 (0.3%)
**Currently using**	6 (1.6%)	–	11 (3%)	–	1 (0.3%)	1 (0.3%)	1 (0.3%)	–	1 (0.3%)	–	4 (1.1%)	–
**Received in Turkey**	6 (1.6%)	–	6 (1.6%)	–	–	1 (0.3%)	–	–	–	–	3 (0.8%)	–
Unmet AP need** (MSI population n = 373)	8 (2.1%)	1 (0.3%)	5 (1.3%)	12 (3.2%)	6 (0.5%)	8 (2.1%)	2 (0.5%)	1 (0.3%)	13 (3.5%)	27 (7.2%)	13 (3.5%)	7 (1.9%)
Coverage***	43%	0%	69%	0%	0%	11%	33%	0%	7%	0%	24%	0%
**Reason do not have AP**												
Need not felt by participant	12.5%	–	80%	8.3%	–	12.5%	–	–	46.2%	22.2%	–	–
Device is broken/unusable	–	–	–	–	–	–	50%	–	7.7%	3.7%	–	–
Didn’t find device helpful	–	–	–	8.3%	–	12.5%	–	–	7.7%	3.7%	–	14.3%
Unaware of available device	–	–	–	16.7%	–	37.5%	–	–	15.4%	29.6%	–	–
Could not afford	37.5%	–	20%	83.3%	66.7%	62.5%	–	100%	15.4%	44.4%	92.3%	71.4%
Service/device not available	75%	–	–	–	50%	25%	50%	100%	46.2%	22.2%	23.1%	42.9%
Transport not accessible	–	–	–	8.3%	–	–	–	–	–	–	–	–
Transport too expensive	12.5%	–	–	8.3%	–	–	–	–	7.7%	3.7%	–	–
Service far away	12.5%	–	–	–	–	–	–	–	–	3.7%	–	–
Negative attitude of service providers	–	100%	–	–	–	–	–	–	7.7%	–	–	–
No translator	–	–	–	–	–	–	–	–	7.7%	3.7%	–	–

*Abbreviations*: *AP* assistive product, *MSI* musculoskeletal impairment, *LL* lower limb, *UL* upper limb, *pros* prosthetic, *orth* orthosis; *Overall need = Need but not using + currently using; **Unmet AP need = Need but do not have; ***Coverage = (currently using) / (Need but not using + currently using)

Current AP use was uncommon for people identified as having MSI: 11 (3%) participants with MSI currently used a stick/cane, six (1.6%) used a wheelchair, and four used a toilet/shower chair (1.1%). For other APs, either one or no participants were currently using. Specifically, in Turkey, the Migrant Health Centre was most commonly accessed for APs.

Unmet need for AP was defined as the proportion of those people with MSI who (according to the physiotherapist) could benefit from a particular AP but were not currently using a particular AP. Overall, 19% (*n* = 70) of people with MSI needed, but were not using, *at least* one AP related to MSI that they could benefit from. Unmet need was highest for protective footwear (27 out of 373, 7.2%) and lower for other APs (see Table 6).

Among people who needed, but were not using an AP, the most common reasons for not using were lack of AP availability (22% to 100%), financial barriers (15% to 100%) and ‘need not felt’ (8% to 80%).

Applying estimates of unmet need, 2.3% (*n* = 70) of the study population of Syrian refugees needed, but were not receiving, *at least* one AP related to MSI that they could benefit from. Extrapolating to the total population of Syrian refugees living in Sultanbeyli suggests there are approximately 500 people who need, but are not be receiving *at least* one MSI related AP.

Coverage for APs, calculated as the proportion of people who are currently using AP out of those who need (but don’t have) or are currently using AP, was very low: there was no coverage for crutches, quad/tripod sticks, protective footwear, upper limb prosthetic and grab bars and less than half for other APs, except walking sticks/canes (69%).

## Discussion

### MSI survey results

This population-based survey of persons aged 2 years and above found that MSI among Syrian refugees living in Sultanbeyli Istanbul was common, with an estimated prevalence of 12.2% of MSI. The prevalence increased significantly by age to 43.8% in adults aged 50 years and older.

Compared to previous studies using the RAM, the prevalence was similar to that found in Cameroon (11.6%) and more than twice the prevalence in Rwanda (5.2%) [13, 15]. The prevalence was lower than the RAM study in India (19.6%) which included an additional screening question on back-pain which may have contributed to the higher estimate [14]. It might also reflect the relatively younger age of the population in the current study where only 8% were > 50 years compared to 19% in India. The prevalence of moderate/severe MSI among Syrian refugees (8.6%) was higher than the three previous RAM studies (India 3.5%, Cameroon 3.4%, Rwanda 2.8%), despite the relatively young age of the current study population [13–15]. This may reflect direct or indirect impact of the Syrian war, such as an injury or challenges in accessing services prior, during or after displacement, leading to more severe impairments. However, it is also possible that this may reflect the revisions made to the RAM survey tool in particular the inclusion of pain in the screening questions and the use of the standardised definitions within the case severity matrix which categorised severity into upper and lower limb and gave classification to severity. For example, a case that could walk the prescribed distance but could not complete this in a given time was described as moderate.

Data on MSI among displaced Syrian populations are lacking for comparison. In the survey conducted with Syrian refugees in Lebanon and Jordan, 14.4% of adults self-reported difficulties walking, similar to the 14.2% prevalence of moderate/severe MSI among adults in our study. However, since this study only used a self-report tool and focussed only on walking, any further comparisons are limited [7].

Our study found that 8% of the Syrian refugee population identified the war in Syria as the cause of their MSI. This proportion is similar to Rwanda, the only other post-conflict population with RAM data, where 4% of the participants reported that their trauma-related MSI occurred during the 1994 genocide, and is higher which is likely due to the differences between the two types of conflict and displacement [13, 19]. Though both findings are of note, they were lower than anticipated. In both settings, it is possible that people were hesitant to cite the Syrian war/Rwanda genocide as the cause of their MSI, leading to under-reporting [19]. To try and mitigate this, the study teams, including the physiotherapists, were either Syrian or from other Arabic speaking countries and we ensured privacy by conducting interviews and examinations in the participants’ homes to encourage more honest and open responses [19]. Additionally, it might also reflect the simplicity of the question given that underlying conditions that may have been exacerbated by the conflict/displacement might not have been recorded. This is consistent with other findings, such as in post-earthquake Haiti where the biggest factor in disability was ageing not the disaster, and further work is needed to explore this finding [20].

Overall need and unmet need for impairment related services among people with MSI was high, particularly for physiotherapy (83%) despite the fact that physiotherapy services are available at the Migrant Health Centre in the district, and coverage was low. Further, nearly a fifth (19%) of people with MSI needed, but were not receiving, at least one AP, and coverage was low amongst those needing AP, except for stick/canes (69%).

Overall these findings suggest a significant gap in access to services and related APs to meet the health, rehabilitation and assistive technology needs for this Syrian population living in Sultanbeyli District. These findings are congruent with limited previous research which suggest widespread barriers to accessing impairment specific services for forced displaced populations [7, 21–23]. For example, a study in Lebanon and Jordon found that 25.5% of Syrian refugees with disabilities were unable to access at least one specialised service despite their needs [7]. Another study among Syrian refugees in Jordan found that forced displacement presented major challenges to people with non-communicable diseases and indicated it was important to continue supporting public sector services to adequately meet their expanding needs [21]. Participants, in our study, reported that lack of availability as well as lack of perceived need and awareness of available services were barriers. Physical rehabilitation services do exist in the community, however are limited and primarily are sought through non-government organisation centres. Therefore, efforts to link people to services and increase both capacity and community awareness of these may be important. Home visits have been found to be important in increasing access to services in other settings [7, 24]. Cost was also a common barrier particularly to accessing APs. This echo’s previous studies and suggests the need for examining fees and social assistance available.

Access to health and rehabilitation services and APs is a human right [5, 25] supported by international humanitarian law [5, 6], and for some people with MSI these interventions can be instrumental for maximising functioning, quality of life and participation in society [9]. People with impairments and disabilities must be consulted about provision of these services and programmes to best meet their needs, especially in humanitarian settings [6]. To respond to this identified gap, service and AP provision should be consultative and comprehensive inclusive of multiple needs (i.e. surgical and post-operative care, medication, rehabilitation and provision of APs) and multiple functional domain needs [7]. It also is essential that comprehensive funding is planned as well for related health and social costs, including transportation to clinic-based services, follow up service visits and maintenance and repair of APs.

### Strengths and limitations

#### Overall survey

This study addresses a gap in MSI data among Syrian refugees and conflict-affected refugee populations more widely. The study used standardised sampling methods and a validated tool. However, limitations exist. The survey response rate was just under 80%. This reflects the complexities of conducting surveys in urban settings and particularly among displaced populations [26]. The recent re-location policies for Syrians in Turkey may have contributed to relatively high (8%) refusals. It is possible that non-responders who were unavailable (i.e. not at home at the time of the survey team visit) were less likely to have had MSI which may have resulted in some over-estimation of the prevalence. However, the age and sex distribution of the study sample was congruent with the migrant registration database. Additionally, the sample was selected from Sultanbeyli Municipality’s refugee registration database so unregistered or undocumented refugees were not included.

#### RAM strengths, limitations and further work

This study was the first to use an updated version of the RAM since it’s validation in Rwanda in 2008 [12]. The addition of the case definitions enabled greater standardisation in the classification of severity and the expanded section on service and AP provided more detailed information on unmet need, coverage and barriers compared to previous RAM surveys.

There are also limitations and areas that could be further developed. First, though the RAM is a structured tool with standardised training and assessment process, the specific diagnosis and needs assessment relies, to some extent, on the clinician’s clinical reasoning and assumptions which are likely influenced by their prior training and may introduce some subjectivity in assessment. For example, the clinicians were physiotherapists and it is possible there was bias resulting in an over-estimation of the need for physiotherapy and under-estimation of other services and APs of which the physiotherapists have less experience. Second, the RAM relies on clinical impairment assessment only, without wider consideration of other factors, such as daily activities, perceived need by the participants and environmental and personal contexts [27], which can be important in determining potential need for some services, such as occupational therapy, and APs, such as ramps. For example, it is noted the primary reason identified for not using services/APs was due to “need not felt” and, given the higher prevalence of MSI in the older age group, there could be other cultural and socio-economic factors that might influence their perceived need. Therefore, future versions of this tool should consider participant perceived need as well as assessment of participant functioning and the environment, and capture the clinicians’ assessment process through the use of clinical decision trees. Third, a significant proportion of aetiologies and diagnoses, in this survey, were originally marked as ‘unknown’ by the physiotherapists which was more than previous surveys. The reasons for this are unclear, but may reflect complexities with those sections, translation issues during training or challenges with filters and skip patterns included in the ODK mobile app for these sections of the tool. Future versions of RAM could be strengthened by inclusion of photographs of different diagnoses to facilitate ease and standardisation of diagnosis. Finally, this was the first study that used tablet-based ODK mobile programming for the RAM as an alternative to paper-based based data collection. Further improvements are needed, particularly in the use of skip patterns, and a bespoke mobile app software with customised built-in features such as skips, filters and photos on a web-based data monitoring platform would improve the tablet-based utility of this tool. With these RAM recommendations, further validation studies would be required.

## Conclusion

MSI is common among the Syrian refugee population living in Sultanbeyli District, particularly among older adults. Further, there is a high unmet need for most MSI-related services and low coverage of both services and APs. These estimates indicate a gap in the current service and AP provision for this displaced refugee population. The findings can be used to inform the planning of migrant health and social services regarding rehabilitation services, provision of APs and initiatives to increase access and uptake of these services to improve functioning and quality of life. This study also identified areas for further development of the RAM tool for musculoskeletal and broader mobility-related impairments.

## Supplementary Information

**Additional file 1.**

**Additional file 2.**

## Data Availability

The dataset used and/or analysed during the current study is available from the corresponding author on reasonable request.

## Abbreviations

AP: Assistive product
GBD: Global Burden of Disease
ICED: International Centre for Evidence in Disability
ICF: International classification of functioning, disability and health
LMIC: Low-and-middle income countries
LSHTM: London School of Hygiene & Tropical Medicine
MSI: Musculoskeletal impairment
ODK: Open Data Kit
RAM: Rapid Assessment of Musculoskeletal impairment
