# Correction to: Musculoskeletal impairment among Syrian refugees living in Sultanbeyli, Turkey: prevalence, cause, diagnosis and need for related services and assistive products

**DOI:** 10.1186/s13031-021-00376-3

**Published:** 2021-06-07

**Authors:** Dorothy Boggs, Oluwarantimi Atijosan-Ayodele, Hisem Yonso, Nathaniel Scherer, Timothy O’Fallon, Gülten Deniz, Selin Volkan, Ahmed Örücü, Isotta Pivato, Ammar Hasan Beck, İbrahim Akıncı, Hannah Kuper, Allen Foster, Andrea Patterson, Sarah Polack

**Affiliations:** 1 International Centre for Evidence in Disability, London School of Hygiene & Tropical Medicine, London, UK; 2 Maidstone and Tunbridge Wells NHS Trust, Maidstone, UK; 3 Relief International, Istanbul, Turkey; 4 Mülteciler Derneği, Istanbul, Turkey; 5 International Centre for Eye Health, London School of Hygiene & Tropical Medicine, London, UK

**Correction to: Confl Health 15, 29 (2021)**

**https://doi.org/10.1186/s13031-021-00362-9**

During the publication process of this original article [1], errors were not amended prior to publication, including an affiliation location, additional file reference and titles, and a few minor omissions and typographical errors in the article. These errors do not affect the scientific content of this article and have been corrected in the original publication.
