# Supplementary material for: Musculoskeletal impairment among Syrian refugees living in Sultanbeyli, Turkey: prevalence, cause, diagnosis and need for related services and assistive products
Source: Confl Health. 2021 Apr 20;15:29. doi: 10.1186/s13031-021-00362-9 (PMC8056489; doi:10.1186/s13031-021-00362-9)
Supplement: Supplementary file 2 — Additional file 2. [file 13031_2021_362_MOESM2_ESM.docx]

**Additional file 2 title: Indicator definitions for services and assistive products proportions calculated**

| **Indicators** | **Services** | **Assistive products (AP)** |
| --- | --- | --- |
| Ever received | Proportion of the MSI cases who previously have received the service. | Proportion of the MSI cases who previously have received the AP. |
| Ever received in Turkey | Proportion of the MSI cases who have received the service in Turkey. | Proportion of the MSI cases who have received the AP in Turkey. |
| Currently accessing/using | Proportion of the MSI cases who are currently accessing the service in Turkey. | Proportion of the MSI cases who are currently using the AP in Turkey. |
| Where currently accessing/using | Proportion of the locations where the MSI cases are currently accessing the service. | Proportion of the locations where the MSI cases are currently using the AP. |
| Unmet need (MSI cases) | Proportion of MSI cases identified as could benefit from receiving the service, but not accessing, the service. | Proportion of MSI cases identified as could benefit from receiving the AP, but not using the AP. |
| Unmet need (survey population) | Proportion of the survey population identified as needing, but not accessing, the service. | Proportion of the survey population identified as needing, but not using the AP. |
| Coverage | Proportion of MSI cases identified who are currently accessing the service in Turkey over those who are currently accessing the service in Turkey and could benefit from receiving the service, but not accessing, the service. | Proportion of the MSI cases who are currently using the AP in Turkey over those who are currently using the AP in Turkey and could benefit from receiving the AP, but not using the AP. |
| Reason not seeking | Proportion of the reasons why the MSI cases who were identified as needing, but not receiving, the service are not accessing the service. | Proportion of the reasons why the MSI cases who were identified as needing, but not receiving, the AP are not accessing the AP. |
